# Statement of Retraction: Methyltransferase-like 14 silencing relieves the development of atherosclerosis via m^6^A modification of p65 mRNA

**DOI:** 10.1080/21655979.2026.2728872

**Published:** 2026-10-01

We, the Editors and Publisher of the journal *Bioengineered*, have retracted the following article from publication. Liu, Y., Luo, G., Tang, Q., Song, Y., Liu, D., Wang, H., & Ma, J. (2022). Methyltransferase-like 14 silencing relieves the development of atherosclerosis via m^6^A modification of p65 mRNA. *Bioengineered*, 13(5), 11832–11843. https://doi.org/10.1080/21655979.2022.2031409 Since publication, significant concerns have been raised by a third party about the integrity of the data and reported results in the article. Specifically, concerns were raised about alleged image duplication with similar images from an unrelated article: ● Figure 4b nuclear p65 band appears to be duplicated with a similar image in Figure 2b CD63/M1 band from Wang et al, 2022 (https://doi.org/10.1080/21655979.2021.2023794).

When approached for an explanation, the authors did not provide their original data or any necessary supporting information and so these serious concerns remain unaddressed. As verifying the validity of published work is core to the integrity of the scholarly record, we are therefore retracting the article. The authors have been informed of this decision. The authors agree with the retraction. We have been informed in our decision-making by our policy on publishing ethics and integrity and COPE guidelines.  The retracted article will remain online to maintain the scholarly record and will be digitally watermarked on each page as ‘Retracted’. 

